# Effects of insomnia and levels of depression and anxiety symptoms on quality of life in people with epilepsy

**DOI:** 10.1186/s12888-022-04154-0

**Published:** 2022-07-25

**Authors:** Rui Zhong, Zhuan Li, Qingling Chen, Hanyu Zhang, Xinyue Zhang, Weihong Lin

**Affiliations:** 1 Department of Neurology, The First Hospital of Jilin University, Changchun, China; 2 Department of Emergency, Linyi Central Hospital, Linyi, China; 3 Department of Hepatology, Second People’s Clinical College of Tianjin Medical University, Tianjin, China

**Keywords:** Insomnia, Depression symptom levels, Anxiety symptom levels, Quality of life, People with epilepsy

## Abstract

**Objectives:**

The association between insomnia and quality of life (QOL) in epilepsy is poorly understood and may involve interactive variables. We aimed to investigate whether and how insomnia, levels of depression and anxiety symptoms interact to influence QOL in people with epilepsy (PWE).

**Methods:**

A consecutive cohort of 179 PWE was enrolled. We collected data on insomnia, levels of depression and anxiety symptoms, and QOL. The Insomnia Severity Index (ISI), Depression Inventory for Epilepsy (NDDI-E), Generalized Anxiety Disorder-7 (GAD-7), and QOL in Epilepsy Inventory (QOLIE-31) were used. The direct, indirect, and total effects of insomnia on QOL were estimated based on a moderated mediation model.

**Results:**

Depression symptom levels mediated the association between insomnia and QOL (B = 0.09 SE = 0.03, *p* = 0.01). Depression symptom levels accounted for 34.7% of the total effect of insomnia on QOL. The mediating effect of depression symptom levels was positively moderated by anxiety symptom levels (B = 0.09, SE = 0.03, *p* = 0.01).

**Conclusion:**

The effect of insomnia on QOL can be partially explained by the mediation of depression symptom levels. Additionally, improving anxiety symptoms may attenuate the indirect effect of insomnia on QOL through depression symptom levels.

## Introduction

Epilepsy is one of the most common serious chronic neurological disorders, affecting more than 70 million patients worldwide [1, 2]. It is characterized primarily by recurrent and unpredictable seizures [3]. PWE often report psychiatric symptoms and sleep disorders such as insomnia [4–6]. Insomnia is the most common sleep-related complaint in epilepsy, and 25% to 54% of patients report insomnia symptoms [7–9]. Insomnia has been known to be associated with a reduced QOL in PWE [9, 10], and PWE with insomnia have significant QOL impairment [11]. However, there is limited evidence on how insomnia influences QOL. Additionally, QOL of PWE are closely associated with the knowledge, attitude, and perceived stigma of the patients [12]. It has also been reported that the QOL was influenced by the disease severity, age, gender, hippocampal sclerosis, number of antiseizure medications (ASMs), and intellectual disability [13–16].

The prior literature has shown that PWE with insomnia generally report more depression symptoms than those without insomnia [9, 17]. Insomnia is positively correlated with depression symptoms [18]. Furthermore, depression has been identified as the most important predictor of poor QOL in PWE [19, 20]. Thus, we speculate that insomnia would increase the levels of depression symptoms, which in turn would reduce QOL. The impacts of insomnia on QOL can be explained by the mediation of depression symptom levels. To our knowledge, no study to date has focused on this point. Additionally, anxiety symptoms were significantly associated with a high level of depression symptoms in PWE [21]. Anxiety was highly dependent on whether the individual had experienced depression [22, 23]. Anxiety and depression are highly comorbid in PWE [24]. High levels of anxiety symptoms are also a strong predictive factor of poor QOL [20]. Thus, we hypothesize that anxiety symptom levels would interact with depression symptom levels to influence the relationship between insomnia and QOL in patients.

This study aimed to investigate the effects of insomnia, levels of anxiety and depression symptom on QOL in PWE. We propose two hypotheses (H): H1: depression symptom levels mediate the association between insomnia and QOL; and H2: anxiety symptom levels moderate the indirect association between insomnia and QOL through depression symptom levels.

## Methods

### Participants and procedures

A cross-sectional study design was employed. Individuals with epilepsy who were treated and followed at the epilepsy center of Jilin University, First Hospital, were consecutively enrolled from April to August 2021. The diagnosis of epilepsy was established by an epileptologist at least one month prior to this study according to the International League Against Epilepsy (ILAE) diagnostic criteria [25]. Other entry criteria were as follows: aged 18 years or older, took stable doses of anti-seizure medications (ASMs) for at least 1 month, and had the ability to understand and complete the questionnaires. We excluded patients who had psychogenic nonepileptic seizures, brain diseases other than epilepsy (e.g., Alzheimer’s disease), serious physical disorders (e.g., cancer) or psychiatric disorders (e.g., schizophrenia). Demographics and clinical variables, including age, sex, age at onset, epilepsy duration, seizure frequency, epilepsy type and ASMs taken, were collected and recorded.

### Questionnaires

Insomnia was assessed using the Insomnia Severity Index (ISI), a self-rated questionnaire of insomnia severity [26]. The ISI was validated in Chinese by Yu et al., and the Cronbach’s α coefficient for the C-NDDI-E was 0.81 [27]. The ISI has 7 items measuring the severity of sleep onset and sleep maintenance difficulties, satisfaction with current sleep, interference with daily functioning, degree of impairment attributed to the sleep issue, and degree of concern caused by the sleep problem. The total score of the ISI ranges between 0 and 28, with a higher score representing greater insomnia severity. A score of 15 or greater on the ISI is the cut point for clinically significant insomnia.

The Depression Inventory for Epilepsy (NDDI-E) was employed to assess depression symptom levels. The NDDI-E is a 6-item measure designed to assess the levels of depression symptoms in PWE [28]. The C-NDDI-E was validated in Chinese by Dong Zhou et al., and the Cronbach’s α coefficient for the C-NDDI-E was 0.825 [29]. The total score ranges between 6 and 24, with a higher score reflecting greater levels of depression symptoms.

The Generalized Anxiety Disorder-7 (GAD-7) was used to assess the levels of anxiety symptoms. The GAD-7 is a brief 7-item screening instrument for the symptoms of generalized anxiety disorder [30]. The Chinese version of the GAD-7 was used to screen the comorbidity of anxiety symptoms in PWE, and the Cronbach’s α coefficient for the GAD-7 was 0.888 [31]. It has a maximum total score of 21, and a higher GAD-7 score indicates higher levels of anxiety symptoms.

Quality of life was measured using the Quality of Life in Epilepsy-31 (QOLIE-31) inventory [32, 33], an epilepsy-specific measure of self-perceived quality of life that consists of 31 items in 7 domains: (1) seizure worry, (2) overall quality of life, (3) emotional well-being, (4) energy/fatigue, (5) cognitive functioning, (6) antiepileptic medication effects, and (7) social functioning. This scale has a score of 0 to 100, with a higher total score denoting a more favorable quality of life. The Chinese version of the QOLIE-31 was validated in Chinese by Ding et al., and the Cronbach’s α coefficient for the QOLIE-31 was 0.898 [34].

### Statistical analysis

Descriptive statistics (i.e., means, SDs, numbers, and percentages) were used to describe the patient’s characteristics. Spearman’s correlation was performed to confirm the correlations between scores of the questionnaires for insomnia, depression and anxiety symptom levels, and QOL. The Mann–Whitney U test was used to compare the levels of depression and anxiety symptoms and QOL between the groups with and without insomnia. We used moderated mediation analysis to test these hypotheses. Mediation analysis was employed to identify whether depression symptom levels mediate the association between insomnia and QOL. The direct, indirect, and total effects of insomnia on QOL were confirmed. Then, moderated mediation analysis was employed to identify whether the mediating associations were moderated by anxiety symptom levels [35]. For all moderated mediation analyses, bootstrap 95% confidence intervals (CIs) were calculated using a bootstrapping approach (with 5000 bootstrap samples) to verify indirect effects. If the 95% confidence intervals did not contain zero, the indirect effect was determined to be statistically significant. All data analyses were performed using SPSS version 26.0 and PROCESS version 3.4, which are SPSS macros to facilitate the estimation of both direct and indirect effects [36].

### Ethics approval and consent to participate

Approval was obtained from the ethics committee of The First Hospital of Jilin University, and all participants provided informed consent. This study complies with the Helsinki Declaration of 1975, as revised in Fortaleza, Brazil 2013.

## Results

A total of 179 patients were eligible for the study, with a median age of 32.56 years and a female percentage of 48.0% (Table 1**).** The correlation analysis results suggested that ISI and NDDI-E scores were positively and moderately correlated (*r* = 0.48, *p* < 0.001), and ISI and QOLIE-31 scores were negatively and moderately correlated (*r* =—0.59, *p* < 0.001) (Table 2). Meanwhile, NDDI-E and QOLIE-31 scores were negatively correlated with a high statistical significance (*r* =—0.61, *p* < 0.001).

**Table 1:** Descriptive characteristics of patients

Characteristics	Mean ± SD or n (%)
Age	32.56 ± 11.73
Gender-female	86 (48.0)
Age at epilepsy onset	25.22 ± 12.91
Epilepsy duration	7.35 ± 8.01
Seizure frequency over the last year	
Seizure-free	53 (29.6)
< 1/month	75 (41.9)
≥ 1/month	51 (28.5)
Epilepsy type	
Focal	146 (81.6)
Generalized	28 (15.6)
Unclassified	5 (2.8)
Polytherapy	58 (32.4)

*SD* Standard deviation

**Table 2:** The relationship between insomnia, depression and anxiety symptoms levels, and QOL

Variables	Insomnia	Depression symptom levels	Anxiety symptom levels	QOL
Insomnia	1			
Depression symptom levels	*r* = 0.48, p < 0.001	1		
Anxiety symptom levels	*r* = 0.52, p < 0.001	*r* = 0.64, p < 0.001	1	
QOL	*r* = —0.59, p < 0.001	*r* = —0.61, p < 0.001	*r* = —0.64, p < 0.001	1

Insomnia = ISI score; Depression symptom levels = NDDI-E score; Anxiety symptom levels = GAD-7 score; QOL = QOLIE-31 score

*QOL* Quality of life

PWE with insomnia presented significantly higher levels of depression symptoms on the NDDI-E score (*p* < 0.001), higher levels of anxiety symptoms on the GAD-7 score (*p* < 0.001) and worse quality of life on the QOLIE-31 total score (*p* < 0.001) than those without insomnia (Table**3**).

**Table 3:** Depression and anxiety symptoms levels, and QOL in PWE with and without insomnia

Variables	Insomnia group (*n* = 23)	Non-insomnia group (*n* = 156)	*P*-value
Depressive symptoms levels	12.00 ± 4.32	7.68 ± 2.47	< 0.001^a^
Anxiety symptoms levels	8.87 ± 4.99	3.21 ± 3.43	< 0.001^a^
QOL	41.83 ± 7.48	54.86 ± 8.95	< 0.001^a^

Depression symptom levels = NDDI-E score; Anxiety symptom levels = GAD-7 score; QOL = QOLIE-31 score

*PWE* People with epilepsy, *QOL* Quality of life

^a^ Mann—Whitney U test

Insomnia had a statistically significant indirect effect on QOL through the mediating variable of depression symptom levels (B =—0.37, SE = 0.08, bootstrap 95% CI =—0.54 to—0.23) (Table 4). The levels of depression symptoms accounted for 34.7% of the total effect of insomnia on QOL. Thus, Hypothesis 1 was supported.

**Table 4:** Total, direct and indirect effect of insomnia on QOL

Effects on QOL	Variables	B	SE	P	Bootstrap 95% CI	
					LL	UL
Total effect (c)	Insomnia	- 1.08	0.11	< 0.001^a^		
Direct effect (c')	Insomnia	- 0.70	0.12	< 0.001^a^		
Indirect effect	a × b	- 0.37	0.08		- 0.54	- 0.23
	Insomnia (a)	0.31	0.04	< 0.001^a^		
	Depression symptom levels (b)	- 1.22	0.21	< 0.001^a^		

Unstandardized regression coefficients are reported. Bold data show statistical significance (*P* < 0.05,or Bootstrap 95% CI does not contain zero)

*LL* Lower limit, *UL* Upper limit, *CI* Confidence interval, *QOL* Quality of life

^a^ Linear regression analysis

**Fig. 1:**
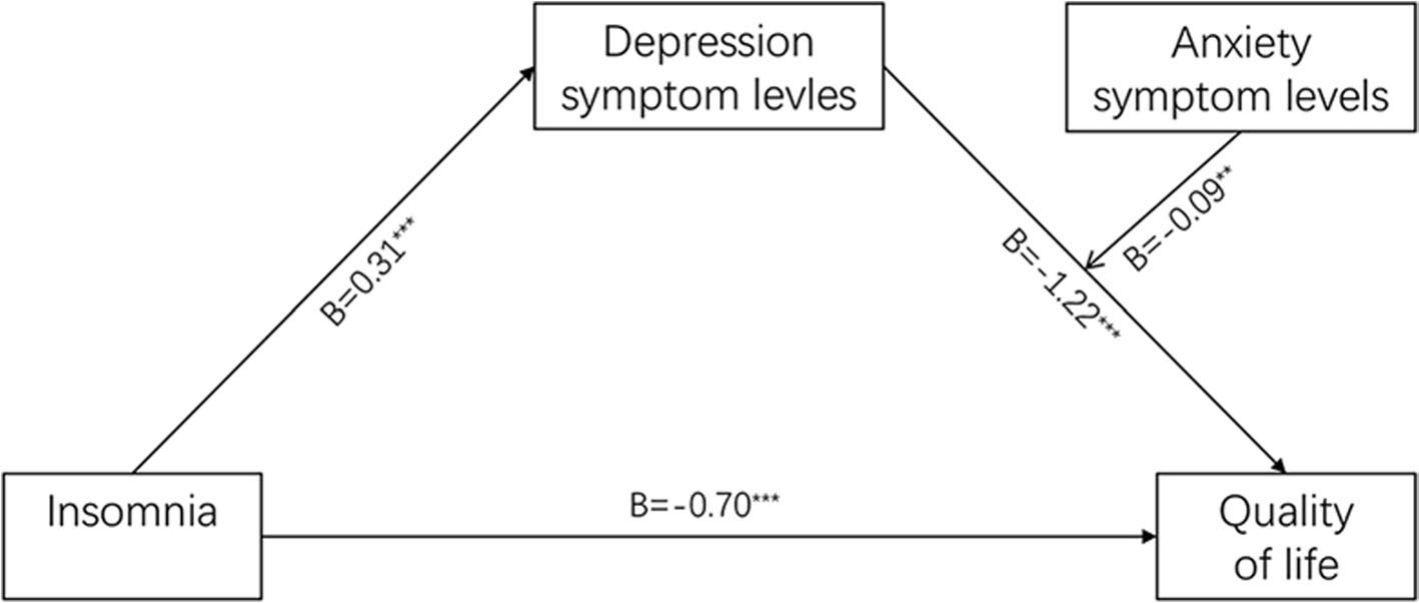
Moderated mediation model. Note. **P < 0.01; ***P < 0.001

The moderated mediation analysis results suggested that the indirect effect of insomnia on quality of life through the mediation of depression symptom levels was positively moderated by anxiety symptom levels (B = 0.09, SE = 0.03, *p* = 0.01). The interaction between depression and anxiety symptoms levels had a significant effect on QOL. The tested model and mean-centered path estimates are displayed in Fig. 1. Therefore, Hypothesis 2 was supported.

## Discussion

We established a moderated mediation model to identify whether the levels of depression symptoms mediate the association between insomnia and QOL and to confirm whether anxiety symptom levels amplify the indirect effect of insomnia on QOL through depression symptom levels. To our knowledge, this is the first exploratory study to investigate the causal pathway between insomnia and QOL through a moderated mediation model. Based on the moderated mediation analysis, we found that the influence of insomnia on QOL can be explained by the mediation of depression symptom levels, and anxiety symptom levels can amplify the indirect effect of insomnia on QOL through depression symptom levels. These results contribute to our understanding of the association between insomnia and QOL.

Our moderated mediation model builds upon prior literature showing the association between sleep disturbances, psychiatric symptoms and QOL [11, 37, 38]. According to the literature, more than half of PWE suffer from insomnia, and their depressive scores are significantly correlated with insomnia, which is in agreement with our results [9]. Additionally, depression represents one of the most common comorbidities in PWE and has a profound negative influence on QOL [39, 40]. Insomnia is significantly associated with poor performance in all seven subscales of the QOLIE-31 [9, 11].

In the established moderated mediation model, we found that insomnia might increase depression symptom levels, which in turn would lead to a reduced QOL. Hence, insomnia had an indirect effect on poor QOL, and depression symptom levels mediate the impacts of insomnia on QOL in PWE. This important finding has some useful clinical implications. From the patients’ perspective, addressing depression symptoms may be just as important as addressing insomnia. For patients with insomnia, interventions targeting this mediator (depression symptom levels), which is associated with QOL, could improve QOL. The conclusion about the indirect association between insomnia and QOL through depression symptom levels uncovers the limitations of conventional regression analysis for understanding the actual interrelationship among risk factors for QOL in PWE [41, 42].

There is a complex relationship between depression and anxiety in the literature [43, 44]. Anhedonia may be a key factor linking these two psychiatric disorders, and anxiety may devolve into depression through anhedonia [45]. The moderating effect of anxiety symptom levels on the association between insomnia and QOL through depression symptom levels was also tested. For the first time, our data showed that the positive association between depression and QOL becomes stronger for patients with higher anxiety symptom levels. Our findings suggest that improving anxiety symptoms may help reduce the impact of depression symptom levels on QOL in PWE.

The current study has some limitations. First, all patients were enrolled from a single center in northeast China; there may exist selection bias, and the results might not be generalizable to the whole country or other countries. Second, our findings were based on a cross-sectional study design, and additional studies are required to replicate our findings and establish causality in prospective longitudinal designs. Third, all patients were taking one or more ASMs, and there may be bias due to the effect of different ASMs on psychiatric symptoms [5, 46]. Additionally, we did not consider the diagnosis of mental disorders and the effects of ASMs. Fourth, the presence of depression and anxiety symptoms were evaluated only with screening tests. Further study using a complete psychiatric evaluation is needed to confirm our findings. Furthermore, insomnia was evaluated using the ISI, a self-rated questionnaire of insomnia severity. However, the evaluation of insomnia without performing polysomnography is always poorly evaluated and with an important scientific bias. Finally, the effects of levels of depression and anxiety symptoms on quality of life may be influenced by other epilepsy-related variables, such as the number of ASMs and seizure frequency, which were not analyzed in our study. Several other aspects besides depression and anxiety symptoms can contribute to the occurrence of insomnia, particularly age and economic and marital aspects, among others, which may influence our established relationship [47, 48]. Future researches are required to confirm our findings with the inclusion of more variables.

## Conclusion

In summary, depression symptom levels mediated the association between insomnia and QOL, and the moderating effect of anxiety symptom levels on this association was also confirmed. The magnitude of this moderated mediation model emphasizes the need to incorporate measures to assess and treat psychiatric conditions, which could improve the QOL in PWE with insomnia.

## Data Availability

The datasets generated and analyzed during the current study are not publicly available because no consent was obtained from the participants in this regard. However, the data are available from the corresponding author upon reasonable request.

## Abbreviations

PWE: People with epilepsy
ASMs: Antiseizure medications
ISI: Insomnia Severity Index
C-NDDI-E: Chinese Version of Neurological Disorders Depression Inventory for Epilepsy
GAD-7: Generalized Anxiety Disorder-7
QOLIE-31: Quality of Life in Epilepsy Inventory
ILAE: International League Against Epilepsy
ORs: Odds ratios
CIs: Confidence intervals

## References

[1] Thijs RD, Surges R, O'Brien TJ, Sander JW (2019). Epilepsy in adults. Lancet.

[2] Saxena S, Li S (2017). Defeating epilepsy: A global public health commitment. Epilepsia Open.

[3] Kanner AM (2016). Management of psychiatric and neurological comorbidities in epilepsy. Nat Rev Neurol.

[4] Kanner AM (2011). Depression and epilepsy: A bidirectional relation?. Epilepsia.

[5] Kanner AM (2003). Depression in epilepsy: prevalence, clinical semiology, pathogenic mechanisms, and treatment. Biol Psychiatry.

[6] Moore JL, Carvalho DZ, St LE, Bazil C (2021). Sleep and Epilepsy: a Focused Review of Pathophysiology, Clinical Syndromes, Co-morbidities, and Therapy. Neurotherapeutics.

[7] Lopez MR, Cheng JY, Kanner AM, Carvalho DZ, Diamond JA, Wallace DM (2013). Insomnia symptoms in South Florida military veterans with epilepsy. Epilepsy Behav.

[8] de Weerd A, de Haas S, Otte A, Trenite DK, van Erp G, Cohen A, de Kam M, van Gerven J (2004). Subjective sleep disturbance in patients with partial epilepsy: a questionnaire-based study on prevalence and impact on quality of life. Epilepsia.

[9] Vendrame M, Yang B, Jackson S, Auerbach SH (2013). Insomnia and epilepsy: a questionnaire-based study. J Clin Sleep Med.

[10] Quigg M, Gharai S, Ruland J, Schroeder C, Hodges M, Ingersoll KS, Thorndike FP, Yan G, Ritterband LM (2016). Insomnia in epilepsy is associated with continuing seizures and worse quality of life. Epilepsy Res.

[11] Piperidou C, Karlovasitou A, Triantafyllou N, Terzoudi A, Constantinidis T, Vadikolias K, Heliopoulos I, Vassilopoulos D, Balogiannis S (2008). Influence of sleep disturbance on quality of life of patients with epilepsy. Seizure.

[12] Yeni K, Tulek Z, Simsek OF, Bebek N (2018). Relationships between knowledge, attitudes, stigma, anxiety and depression, and quality of life in epilepsy: A structural equation modeling. Epilepsy Behav.

[13] Radovic NI, Bozic K, Duric AP, Vodopic S, Radulovic L, Vujisic S (2017). Health-related quality of life in adolescents with epilepsy in Montenegro. Epilepsy Behav.

[14] Tedrus G, Crepaldi CR, de Almeida FB (2020). Quality of life perception in patients with epilepsy for a period of 4years. Epilepsy Behav.

[15] Zamani G, Shiva S, Mohammadi M, Mahmoudi GJ, Rezaei N (2014). A survey of quality of life in adolescents with epilepsy in Iran. Epilepsy Behav.

[16] Snoeijen-Schouwenaars FM, van Ool JS, Tan IY, Aldenkamp AP, Schelhaas HJ, Hendriksen J (2019). Mood, anxiety, and perceived quality of life in adults with epilepsy and intellectual disability. Acta Neurol Scand.

[17] Jo S, Kim HJ, Kim HW, Koo YS, Lee SA (2020). Sex differences in factors associated with daytime sleepiness and insomnia symptoms in persons with epilepsy. Epilepsy Behav.

[18] Lee SA, Jung M, Kim SJ, Jo S, Kim HJ, Kim HW, Koo YS (2020). Insomnia is less prevalent and less severe, independent of depressive symptoms, in patients with epilepsy treated with perampanel as an adjuvant. Epilepsy Behav.

[19] Chen YY, Huang S, Wu WY, Liu CR, Yang XY, Zhao HT, Wu LC, Tan LZ, Long LL, Xiao B (2018). Associated and predictive factors of quality of life in patients with temporal lobe epilepsy. Epilepsy Behav.

[20] Zhong R, Lu Y, Chen Q, Li M, Zhao Q, Zhang X, Lin W (2021). Sex differences in factors associated with quality of life in patients with epilepsy in Northeast China. Epilepsy Behav.

[21] Wei Z, Ren L, Wang X, Liu C, Cao M, Hu M, Jiang Z, Hui B, Xia F, Yang Q (2021). Network of depression and anxiety symptoms in patients with epilepsy. Epilepsy Res.

[22] Saade YM, Nicol G, Lenze EJ, Miller JP, Yingling M, Wetherell JL, Reynolds CR, Mulsant BH (2019). Comorbid anxiety in late-life depression: Relationship with remission and suicidal ideation on venlafaxine treatment. Depress Anxiety.

[23] Bauer EA, MacNamara A: Comorbid anxiety and depression: Opposing effects on the electrocortical processing of negative imagery in a focal fear sample. Depress Anxiety 2021. 10.1002/da.23160 PMC8640943 33909324

[24] Jackson MJ, Turkington D (2005). Depression and anxiety in epilepsy. J Neurol Neurosurg Psychiatry.

[25] Fisher RS, Acevedo C, Arzimanoglou A, Bogacz A, Cross JH, Elger CE, Engel JJ, Forsgren L, French JA, Glynn M (2014). ILAE official report: a practical clinical definition of epilepsy. Epilepsia.

[26] Bastien CH, Vallieres A, Morin CM (2001). Validation of the Insomnia Severity Index as an outcome measure for insomnia research. Sleep Med.

[27] Yu DS (2010). Insomnia Severity Index: psychometric properties with Chinese community-dwelling older people. J Adv Nurs.

[28] Gilliam FG, Barry JJ, Hermann BP, Meador KJ, Vahle V, Kanner AM (2006). Rapid detection of major depression in epilepsy: a multicentre study. Lancet Neurol.

[29] Tong X, An D, Lan L, Zhou X, Zhang Q, Xiao F, Park SP, Kanemoto K, Kanner AM, Zhou D (2015). Validation of the Chinese version of the Neurological Disorders Depression Inventory for Epilepsy (C-NDDI-E) in West China. Epilepsy Behav.

[30] Spitzer RL, Kroenke K, Williams JB, Lowe B (2006). A brief measure for assessing generalized anxiety disorder: the GAD-7. Arch Intern Med.

[31] Tong X, An D, McGonigal A, Park SP, Zhou D (2016). Validation of the Generalized Anxiety Disorder-7 (GAD-7) among Chinese people with epilepsy. Epilepsy Res.

[32] Cramer JA, Perrine K, Devinsky O, Bryant-Comstock L, Meador K, Hermann B (1998). Development and cross-cultural translations of a 31-item quality of life in epilepsy inventory. Epilepsia.

[33] Saadi A, Patenaude B, Mateen FJ (2016). Quality of life in epilepsy-31 inventory (QOLIE-31) scores: A global comparison. Epilepsy Behav.

[34] Hu Y, Guo Y, Wang YQ, Du Q, Ding MP (2009). Reliability and validity of a Chinese version of the Quality of Life in Epilepsy Inventory (QOLIE-31-P). Zhejiang Da Xue Xue Bao Yi Xue Ban.

[35] Hayes AF (2015). An Index and Test of Linear Moderated Mediation. Multivariate Behav Res.

[36] Preacher KJ, Hayes AF (2004). SPSS and SAS procedures for estimating indirect effects in simple mediation models. Behav Res Methods Instrum Comput.

[37] Camara-Lemarroy CR, Hoyos M, Ibarra-Yruegas BE, Diaz-Torres MA, De Leon R (2017). Affective symptoms and determinants of health-related quality of life in Mexican people with epilepsy. Neurol Sci.

[38] Macedo P, Oliveira PS, Foldvary-Schaefer N, Gomes M (2017). Insomnia in people with epilepsy: A review of insomnia prevalence, risk factors and associations with epilepsy-related factors. Epilepsy Res.

[39] Kanner AM (2009). Depression and epilepsy: a review of multiple facets of their close relation. Neurol Clin.

[40] Luoni C, Bisulli F, Canevini MP, De Sarro G, Fattore C, Galimberti CA, Gatti G, La Neve A, Muscas G, Specchio LM (2011). Determinants of health-related quality of life in pharmacoresistant epilepsy: results from a large multicenter study of consecutively enrolled patients using validated quantitative assessments. Epilepsia.

[41] Baranowski CJ (2018). The quality of life of older adults with epilepsy: A systematic review. Seizure.

[42] Siarava E, Hyphantis T, Katsanos AH, Pelidou SH, Kyritsis AP, Markoula S (2019). Depression and quality of life in patients with epilepsy in Northwest Greece. Seizure.

[43] Tiller JW (2013). Depression and anxiety. Med J Aust.

[44] Cummings CM, Caporino NE, Kendall PC (2014). Comorbidity of anxiety and depression in children and adolescents: 20 years after. Psychol Bull.

[45] Winer ES, Bryant J, Bartoszek G, Rojas E, Nadorff MR, Kilgore J (2017). Mapping the relationship between anxiety, anhedonia, and depression. J Affect Disord.

[46] Dreier JW, Pedersen CB, Gasse C, Christensen J (2019). Antiepileptic Drugs and Suicide: Role of Prior Suicidal Behavior and Parental Psychiatric Disorder. Ann Neurol.

[47] Ahmed AE, Al-Jahdali H, Fatani A, Al-Rouqi K, Al-Jahdali F, Al-Harbi A, Baharoon S, Ali YZ, Khan M, Rumayyan A (2017). The effects of age and gender on the prevalence of insomnia in a sample of the Saudi population. Ethn Health.

[48] Kawata Y, Maeda M, Sato T, Maruyama K, Wada H, Ikeda A, Tanigawa T (2020). Association between marital status and insomnia-related symptoms: findings from a population-based survey in Japan. Eur J Public Health.

